# Persistent hypoxemia and platypnea-orthodeoxia after left single-lung transplantation: a case report

**DOI:** 10.1186/s13256-015-0598-4

**Published:** 2015-06-12

**Authors:** Hamza Salim, Jose Melendez, Harish Seethamraju

**Affiliations:** 1 Department of Biochemistry, University of Houston, 4800 Calhoun Rd., Houston, TX 77004 USA; 2 Memorial Hermann System/ Fellowship at Baylor College of Medicine, Memorial Hermann Northwest Hospital, 1635 North Loop Houston, TX 77008. Baylor College of Medicine, 1 Baylor Plaza, Houston, TX 77030 USA; 3 Lexington, KY (Private Practice). Formerly at Methodist Hospital’s JC Walter Jr Lung Transplant Center/Fellow at Baylor College of Medicine, Houston Methodist Hospital 6565 Fannin Street, Houston, TX 77030. 740 S Limestone A301, Lexington, KY 40536 USA

## Abstract

**Introduction:**

Platypnea-orthodeoxia is a relatively uncommon but striking clinical syndrome characterized by dyspnea and deoxygenation accompanying a change to sitting or standing from a recumbent position. Hypoxemia early after lung transplant can have multiple etiologies. We report a rare case of persistent hypoxemia and platypnea-orthodeoxia after left single-lung transplantation, as a result of right-to-left interatrial shunt through a patent foramen ovale, with subsequent resolution of hypoxemia after percutaneous closure of the patent foramen ovale.

**Case presentation:**

Our 66-year-old Caucasian male patient exhibited a persistent patent foramen ovale. Persistent patent foramen ovale produces an intermittent intra-atrial right-to-left shunt and occurs in approximately 25 % of the general population. Although the majority of people with patent foramen ovale are asymptomatic, it is believed to act as a pathway for chemicals or thrombi that can result in a variety of clinical manifestations, including stroke, migraine headache, decompression sickness, high-altitude pulmonary edema, and platypnea-orthodeoxia syndrome. Percutaneous closure of the patent foramen ovale has been shown to be effective in the case of right-to-left shunting with normal pulmonary arterial pressure, but the indication remains controversial in other situations where pulmonary pressures are not normal. The most common causes of hypoxemia immediately after lung transplant include: graft dysfunction, reperfusion injury, acute thromboembolic disease, and acute rejection. We report a case of reopening of a patent foramen ovale after left single-lung transplantation with normal pulmonary pressure.

**Conclusions:**

Our case demonstrates that an open patent foramen ovale leading to massive right-to-left shunting is a possible complication after lung transplant, with significant morbidity, and that it can be treated successfully using a percutaneously placed occlusion device. Through this case report, we aim to improve pre-transplant procedures by demonstrating that a bubble contrast transesophageal echocardiogram can be performed pre-operatively to detect a patent foramen ovale.

## Introduction

We present the case of a 66-year-old Caucasian man with idiopathic pulmonary fibrosis, on three liters of oxygen supplementation, who presented to our institution for a lung transplantation. He was previously evaluated at another transplant center, but he was not considered to be a candidate due to gastroesophageal reflux with mild gastric dysmotility. He was seen at our center for a second opinion and was accepted for transplantation. His medical history included benign prostate hypertrophy, hyperlipidemia, and heart failure (New York Heart Association Class 3) due to his severe deconditioned pre-transplant state as a result of severe lung disease. His surgical history showed that he had undergone an appendectomy when he was 19-years-old without complications.

## Case presentation

In the pre-transplant work-up done prior to our evaluation, his transthoracic echocardiogram (TTE) showed normal left ventricular systolic function, and a right heart catheterization revealed mild pulmonary hypertension (mean pulmonary artery pressure 28mmHg) with a normal cardiac index. In addition, a left heart catheterization was performed and was not indicative of coronary artery disease, with normal O_2_ saturations. His lung perfusion scan showed no evidence of extra-respiratory fixation of technetium. Partial pressures of oxygen (PaO_2_) and carbon dioxide (PaCO_2_) on room air at evaluation were 62mmHg and 46mmHg, respectively. His pulmonary function tests showed a spirometry diffusion capacity of 30 % predicted and severe restriction. A computed tomography (CT) scan of his chest revealed extensive bilateral diffuse pulmonary fibrosis, with severe honeycombing. His ventilation-perfusion (V/Q) scan showed no evidence of extra-respiratory fixation of technetium, and also a low probability of pulmonary embolism. He underwent a left single-lung transplant off-pump. The cytomegalovirus status was donor and recipient positive.

**Fig. 1:**
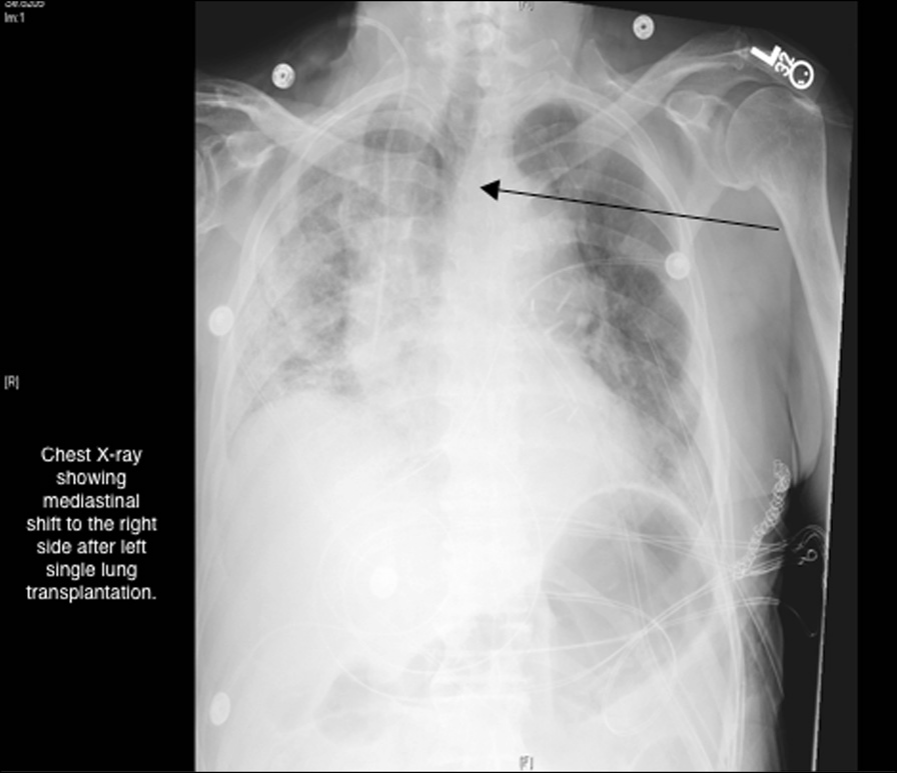
Post-Operative Chest X-ray Indicating a Mediastinal Shift. Arrow points to mediastinal shift to the right side after left lung transplantation

The intra-operative and early post-operative periods were uneventful, allowing extubation on post-operative day (POD) one, and discharge from the intensive care unit (ICU) at POD four. Post-extubation, he still required a significant amount of supplemental oxygen (5 liters/min) to keep his oxygen saturation level at around 90 %, higher even than the amount required in the pre-operative period. His chest X-ray showed a normal left lung and a fibrosed right lung, with a mediastinal shift to the right side (Fig. 1). His bronchoscopy revealed normal left bronchial anastomosis with patent distal airways. We performed a bronchioloalveolar lavage, which was negative for bacterial, viral, and fungal agents. The results of his transbronchial biopsies were negative for acute rejection. A contrast-enhanced CT of his chest did not disclose any sign of pulmonary embolism, and showed minimal atelectasis in the lower lobe of the transplanted lung.

**Fig. 2:**
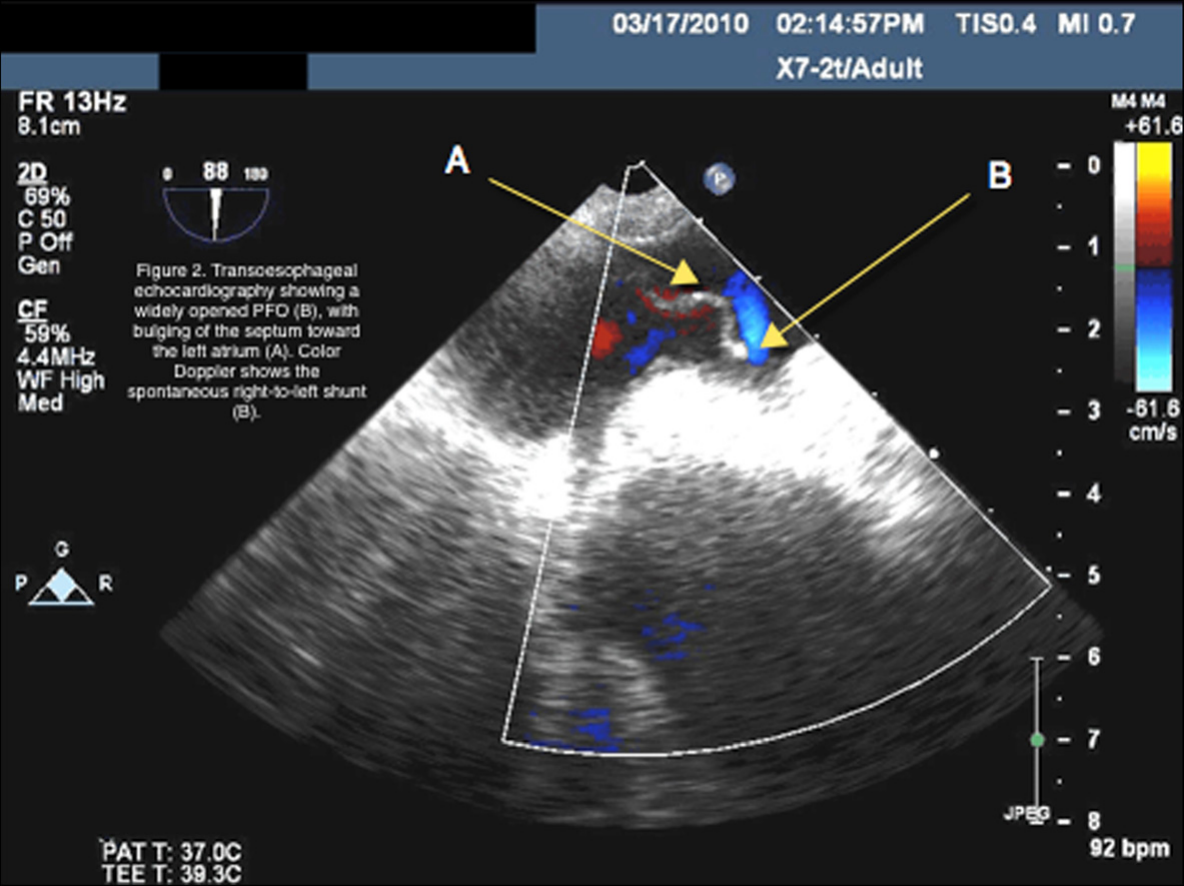
Post-Operative Transesophageal Echocardiogram reveals Widely opened PFO. Arrow A points to the bulging of the septum toward the left atrium. Arrow B points to the color Doppler showing the spontaneous right-to-left shunt

On POD seven, he developed a new complaint of worsening dyspnea in an upright position or walking, which was relieved by lying in a supine position. This was associated with desaturation on pulse oxymetry (platypnea-orthodeoxia). A V/Q scan was ordered which showed a perfusion to the right lung of 80 %, normal ventilation to the transplanted lung, and significant tracer uptake by the kidneys and brain, indicative of a right-to-left shunt. We performed a two-dimensional TTE, which showed normal pulmonary pressure and left ventricular ejection fraction. The main abnormality was an aneurysm motion defect of the inter-atrial septum (Fig. 2). His color Doppler and saline contrast echocardiogram tests showed a patent foramen ovale (PFO) with massive spontaneous right-to-left shunting. The aortic root was also seen to be dilated (4.0cm).

As his respiratory condition had deteriorated in the week following the transplant, he underwent an emergent percutaneous PFO closure. A large PFO (7mm in diameter) with a massive right-to-left shunt was confirmed (Fig. 2). Right-side pressures were normal on his right heart catheterization (mean 16). The PFO was closed using a 35-mm occluding device (Amplatzer™ PFO Occluder, AGA Medical Corporation, Minnesota, USA) with ultrasound-guided transesophageal echocardiography. No residual shunt was observed immediately after implantation. Immediately after the procedure, his oxygen saturation improved with minimal oxygen supplementation. The day after the procedure, he was eupneic at rest, with a PaO_2_ and PaCO_2_ of 82mmHg and 34mmHg, respectively, on room air. He was discharged on POD 12. His respiratory status remains satisfactory 10 months after his lung transplant, and he has no need for supplemental oxygen.

## Discussion

We present this rare case of marked hypoxemia with platypnea-orthodeoxia and significant deterioration of gas exchange after a left single-lung transplant due to right-to-left inter-arterial shunting through a PFO. The PFO either existed before the transplant, or more likely, opened after his transplant surgery [1]. It is our understanding that the PFO became evident after the transplant due to the anatomical changes the transplant brought about. His pre-transplant evaluation did not include a contrast-enhanced TTE, though his perfusion scan did not show evidence of a right-to-left shunt. At our center, PFO is checked for pre-operatively on a routine basis by means of contrast-enhanced TTE and shunting views on a V/Q scan. After ruling out all other causes, we can confidently say that the post-operative right-to-left inter-arterial shunt was the only cause of our patient’s severe life-threatening hypoxemia. To the best of our knowledge, this is the second reported case in the English literature of severe hypoxemia after lung transplant due to right-to-left inter-arterial shunting with normal right-sided pressures. Thabut *et al*. reported the first case, in which the patient presented with delayed PFO reopening on POD 16 after left lung transplant [2].

The pathogenesis of the worsening of a right-to-left shunt through a large PFO after a left single-lung transplant is usually due to elevation of pressure in the right side of the heart [3]. However, in our patient, his right-side cardiac pressure was normal. Also, his CT angiogram did not show significant pulmonary embolism or vascular anastomoses abnormalities. The underlying mechanism can be explained by what has been described in cases of platypnea-orthodeoxia syndrome observed after pneumonectomy [4–6], or in patients with an enlarged aortic root, like ours [7–9]. Platypnea-orthodeoxia syndrome, characterized by the association of dyspnea and hypoxemia aggravated in the upright position and relieved in the supine position, is related to intracardiac or intrapulmonary right-to-left shunting of various causes. The described hypothesis for the mechanism of the reopening of the PFO is the deviation of the mediastinum toward the right side, which occurs after right pneumonectomy, leading to an orientation of the inferior vena cava flow toward the PFO. In terms of the aortic root dilation, an anatomical change of the atrial septum is described that is related to the pressure applied from the dilated aortic root down to the atrium, causing an aneurismal motion defect [10].

The redirection of flow, caused by an anatomic distortion of the right atrium or atrial septum plus the aortic root dilation, is also part of the hypothesis of the cause of platypnea-orthodeoxia after pneumonectomy [7, 8]. In the case of our patient, we can hypothesize that the left transplanted lung, with higher volume compared with the contralateral fibrotic right lung, to some extent will lead to a mediastinal shift, causing distortion of the vena cava and mimicking what is observed after a right pneumonectomy [11].

The mechanism of the reopening of the PFO after lung transplant with normal pulmonary pressure is still unknown, but regardless of the mechanism, this should be considered as a potential complication after lung transplant surgery especially left single-lung transplant. Right-to-left shunting through a PFO should be discussed and evaluated if hypoxemia is not explained by the usual causes (such as infection, rejection, pulmonary embolism, and so on), particularly when the chest radiograph is normal or near normal [11]. Whether to screen for PFO in the pre-transplant evaluation is an unsolved issue, but our observations suggest that, given the potential subsequent complication, a systematic pre-operative PFO search by means of a bubble contrast transesophageal cardiogram may be useful, at least in the case of single-lung transplantation.

## Conclusions

In conclusion, our case demonstrates that an open PFO leading to massive right-to-left shunting is a possible complication after a lung transplant, with significant morbidity, and that it can be treated successfully using a percutaneously placed occlusion device.

## Consent

Written informed consent was obtained from the patient for publication of this case report and any accompanying images. A copy of the written consent is available for review by the Editor-in-Chief of this journal.

## Abbreviations

CT: Computed tomography
PaCO_2_: Partial pressure of carbon dioxide
PaO_2_: Partial pressure of oxygen
PFO: Patent foramen ovale
POD: Post-operative day
TTE: Transthoracic echocardiogram
V/Q: Ventilation/perfusion

## References

[1] Guerin P, Lambert V, Godart F, Legendre A, Petit J, Bourlon F, et al. Transcatheter closure of patent foramen ovale in patients with platypnea–orthodeoxia: results of a multicentric French registry. Cardiovasc Intervent Radiol. 2005;28:164–8. 10.1007/s00270-004-0035-3 15719178

[2] Thabut MDM, Fournier MD, Wolff MDM. Delayed reopening of a hemodynamically significant patent foramen ovale after left lung transplantation: emergency management. J Heart Lung Transplant. 2010;29(4):224–7. 10.1016/j.healun.2009.09.009 19944629

[3] Meier B, Lock JE. Contemporary management of patent foramen ovale. Circulation. 2003;107:5–9. 10.1161/01.cir.0000046073.34261.c1 12515733

[4] Godart F, Rey C, Prat A, Vincentelli A, Chmaït A, Francart C, et al. Atrial right-to-left shunting causing severe hypoxaemia despite normal right-sided pressures: report of 11 consecutive cases corrected by percutaneous closure. Eur Heart J. 2000;21:483–9. 10.1053/euhj.1999.1944 10681489

[5] Bakris NC, Siddiqi AJ, Fraser Jr CD, Mehta AC. Right-to-left interatrial shunt after pneumonectomy. Ann Thorac Surg. 1997;63:198–201. 10.1016/s0003-4975(96)01015-6 8993265

[6] Godart F, Porte HL, Rey C, Lablanche JM, Wurtz A. Postpneumonectomy interatrial right-to-left shunt: successful percutaneous treatment. Ann Thorac Surg. 1997;64:834–6. 10.1016/s0003-4975(97)00607-3 9307484

[7] Smeenk FW, Postmus PE. Interatrial right-to-left shunting developing after pulmonary resection in the absence of elevated right-sided heart pressures: review of the literature. Chest. 1993;103:528–31. 10.1378/chest.103.2.528 8432148

[8] Bellato V, Brusa S, Balazova J, Marescotti S, De Carla D, Bordone G. Platypnea–orthodeoxia syndrome in interatrial right to left shunt postpneumonectomy. Minerva Anestesiol. 2008;74:271–5. 18356806

[9] Eicher JC, Bonniaud P, Baudouin N, Petit A, Bertaux G, Donal E, et al. Hypoxaemia associated with an enlarged aortic root: a new syndrome? Heart. 2005;91:1030–5. 10.1136/hrt.2003.027839 PMC1769048 15761046

[10] Soriano CJ, Balaguer JR, Lluch A, Pérez-Boscá JL, Pomar F, Sanchez J, et al. Right-to-left interatrial shunt despite normal pulmonary artery pressure: anatomical implications. Int J Cardiol. 2006;113:422–4. 10.1016/j.ijcard.2005.08.073 16325282

[11] Pemberton J, Irvine T, Stewart MJ, Antunes G, Gibson GJ. Platypnoea orthodeoxia in a patient with aortic root dilatation and a patent foramen ovale. Eur J Echocardiogr. 2007;8:151–4. 10.1016/j.euje.2005.12.010 16451829

